# Measurement of the uterocervical angle for the prediction of preterm birth in symptomatic women

**DOI:** 10.1007/s00404-021-06002-0

**Published:** 2021-03-05

**Authors:** Philipp Wagner, Jana Schlechtendahl, Markus Hoopmann, Natalia Prodan, Harald Abele, Karl Oliver Kagan

**Affiliations:** Department of Obstetrics and Gynaecology, University of Tuebingen, Calwerstrasse 7, 72076 Tübingen, Germany

**Keywords:** Preterm birth, Cervical length, Preterm labor, Uterocervical angle

## Abstract

**Purpose:**

To examine if the uterocervical angle (UCA) can be used to predict preterm delivery in women with painful and regular uterine contractions and a cervical length of 25 mm or less.

**Methods:**

Retrospective study at the perinatal unit of the University Hospital of Tuebingen, Germany. Women with singleton gestation and preterm contractions between 24 + 0 and 33 + 6 weeks’ gestation were included. For the UCA measurement, a line is placed from the internal os to the external os irrespective of whether the cervix is straight or curved. A second line is drawn to delineate the lower uterine segment. The angle between the two lines is the UCA measurement. The measurements were taken on stored images from our database.

**Results:**

The study consisted of 213 singleton pregnancies. At the time of UCA measurement, median maternal and gestational age was 31.4 years and 29.7 weeks’ gestation. Median gestational age at delivery was 35.3 weeks and the corresponding birth weight 2480 g, respectively. The UCA measurement in women who delivered within 2 days, between 3–7 days and after 7 days was not helpful to distinguish between these three groups [median UCA measurements: 108.5°, 108.0° and 107.3° (Kruskal–Wallis test *p* = 0.576)]. Uni- and multivariate logistic multiple regression analysis demonstrated that the delivery within 2 days was only dependent on the gestational age and the cervical length at the time of presentation.

**Conclusion:**

The measurement of UCA is not useful in predicting preterm birth in the subsequent 7 days after an episode of preterm contractions.

## Introduction

Prematurity is one of the major causes for perinatal morbidity, mortality, and lifelong impairments [1]. Approximately 15 million premature babies are born worldwide every year. However, the diagnosis of premature labor remains challenging. Of the women presenting with symptoms of preterm delivery, only 10–15% will deliver within the next 2 to 7 days [2].

Numerous work groups have investigated biophysical and biochemical methods that could be used to distinguish between true and false labor [3]. Today, cervical length assessment belongs to the standard of care in women with threatened preterm labor [4–6]. Sortiriades et al. examined the usefulness of measuring the cervical length in these women and reported on a detection rate of about 60% for a delivery within the next 7 days for a false positive rate of 9.5% [7]. In a meta-analysis from Berghella et al., the authors summarized the findings of three randomized studies that stratified the further management of women with preterm labor according to the cervical length [8]. In the group of women with known cervical length measurement, the preterm delivery rate was 22% while it was 35% in the group without cervical length information [8]. Similarly, the authors of a recent Cochrane analysis concluded that the knowledge of the cervical length may lead to a prolongation of pregnancy by about 4 days [9].

Although measurement of the length of the cervical is considered standard, effort has been made to find alternative or additive methods to examine the cervix. Dziadosz et al. investigated whether the uterocervical angle (UCA) could be useful for the prediction of preterm birth [10]. For the measurement, the rays were placed on the cervical canal and on the lower uterine segment. The authors reported on a positive correlation between the width of the angle and the risk of preterm delivery. In their study, the detection rate in screening for preterm delivery in the second trimester was far better than with the cervical length [10]. Daskalakis et al. [11] summarized the currently limited knowledge about the UCA. Based on 11 studies, the authors concluded that the UCA measurement might be useful as a predictive factor of PTB [11]. So far, these studies have focused on screening for preterm delivery in the second trimester but not in women with preterm contractions.

In this study, we set out to examine, if the UCA can be used to predict preterm delivery in women with preterm contractions.

## Material and methods

This is a retrospective study, which was performed in the perinatal unit at the University Hospital of Tuebingen, Germany between 2012 and 2018. All women with singleton gestation who presented with painful and regular uterine contractions and a cervical length of 25 mm or less at 24 + 0 to 33 + 6 weeks of gestation were included. Women with ruptured membranes, history of cervical conization, those who had a cerclage placed in the current pregnancy, and those with cervical dilatation were excluded from the study. Some of the patients of in the present study were also included in our previous study on cervical length and preterm delivery [12].

Our perinatal unit is one of the largest tertiary referral centers in Germany with 3500 deliveries per year. Our standard management of women with singleton pregnancies who are suspected to be in preterm labor includes a transvaginal measurement of the cervical length by an experienced obstetrician, administration of tocolytics (usually oral nifedipine) for no more than 48 h, and administration of steroids for pulmonary maturity. Antibiotics are administered only if an ascending infection is suspected or if the cervical length is less than 5 mm and group B streptococcus status of the patient woman is unknown.

The patients were retrospectively identified by searching through our digital perinatal database.

The following data were recorded: maternal age and parity, gestational age at presentation and at delivery, detailed pregnancy history including history of preterm birth, and relevant pregnancy complication. Pregnancy outcome data were obtained from the same database. The cervical length measurements that were obtained using transvaginal ultrasound as well as the presence or absence of funneling and sludge were also recorded. Ultrasound data were obtained from our digital ultrasound database (Viewpoint, GE Healthcare, Munich/Germany).

**Fig. 1:**
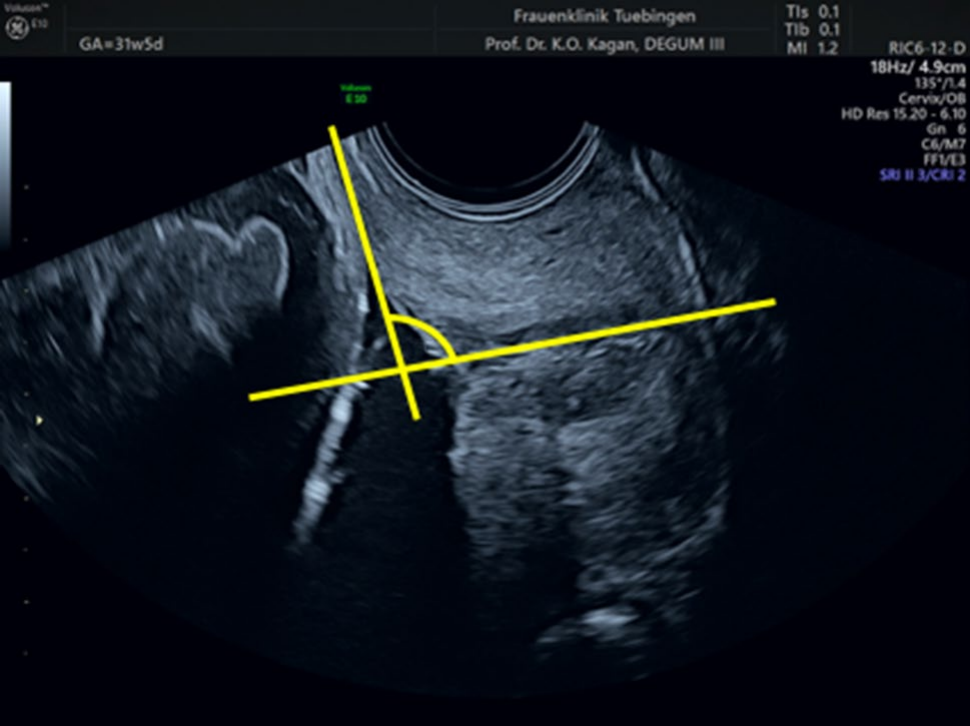
Measurement of the uterocervical angle (UCA) in a women with a cervical length of 22 mm. The arrow highlights the angle of interest

The UCA was measured according to the method described by Dziadosz et al. [10]: in short, a first line is placed from the internal os to the external os irrespective of whether the cervix is straight or curved. The calipers are placed where the anterior and posterior walls of the cervix touch the internal and external os along the endocervical canal. A second line is then drawn to delineate the lower uterine segment. This ray is traced up the anterior uterine segment to a distance allowed by the preloaded image. Ideally, the second ray reaches 3 cm up the lower uterine segment to establish an adequate measurement. The angle between the two lines is the UCA measurement (Fig. 1).

**Fig. 2:**
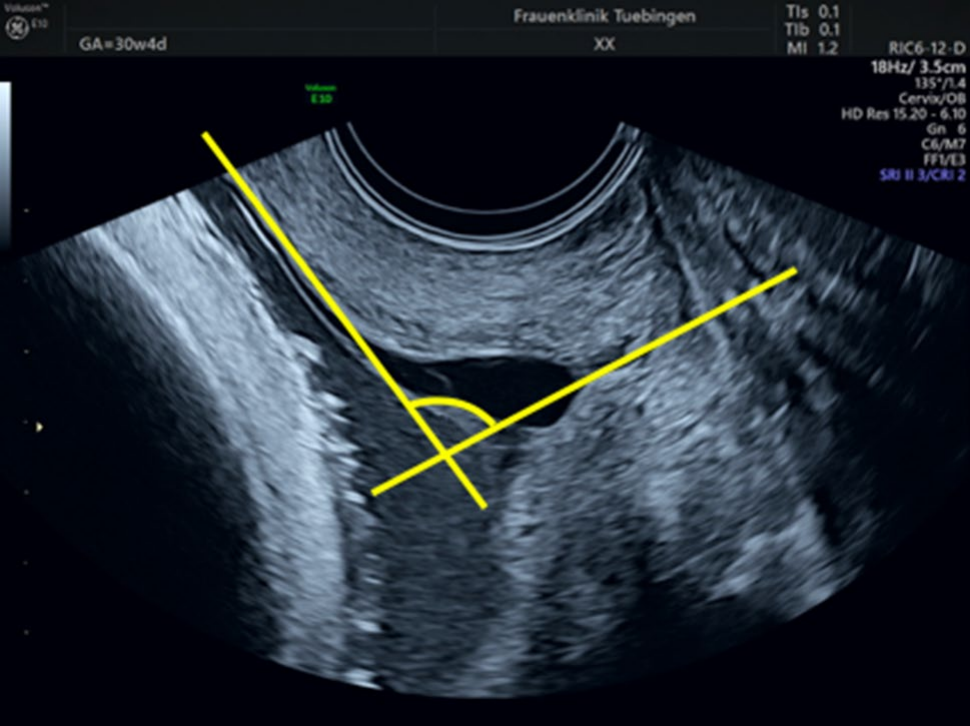
Measurement of the uterocervical angle (UCA) in a women with a cervical length of 10 mm and funneling. The arrow highlights the angle of interest

In the presence of funneling, the first line is placed in the same way as if the cervical length is measured. The second caliper is placed tangentially on the lower uterine segment and extended toward the first line [10] (Fig. 2).

The measurements were performed with Osirix Lite 11.0 (Bernex, Switzerland).

The study was approved by our ethical committee of the University of Tuebingen (No. 041/2020BO2).

### Statistical analysis

The study cohort was clustered into three groups: delivery within 2 days, between 3–7 days and after 7 days after admission due to painful contractions.

For each group, we calculated the median UCA, cervical length and the proportion of cases with funneling and sludge. The differences between the groups were tested with a Kruskal–Wallis or an ANOVA-test whatever was suitable after the Kolmogorov–Smirnov and Levene test for normal distribution and homogeneity of variances. Uni- and multivariate regression analysis was used to identify significant covariates with UCA.

Descriptive data is given as median and 25–75th interquartile range (IQR) or percentage. A *p* value of 0.05 was used as significance level.

## Results

The search of the digital database identified 217 women with singleton pregnancies who were seen due to preterm contractions. In all cases, the cervical length was 25 mm or less. 4 pregnancies were excluded from the further analysis because the stored ultrasound image did not show the lower uterine segment. Thus, our study population consisted of 213 singleton pregnancies.

At the time of UCA measurement, median maternal and gestational age was 31.4 years and 29.7 weeks’ gestation. Median gestational age at delivery was 35.3 weeks and the corresponding birth weight was 2480 g, respectively.

The median time interval between the measurement of the UCA and delivery was 36 days. 25 (11.7%) women delivered within 2 days, 21 (9.9%) within 3–7 days and 167 (78.4%) after 7 days, respectively.

Further details of the study population are shown in Table 1.

**Table 1:** Study population stratified according to the time interval between UCA measurement and delivery

	Delivery ≤ 2 days	Delivery 3–7 days	Delivery > 7 days
	*n* = 25	*n* = 21	*n* = 167
Maternal age Median (IQR)	34.5 (28.6–37.0)	31.6 (27.6–35.5)	31.1 (27.7–34.8)
Gestational age Median (IQR)	31.1 (28.4–32.7)	30.4 (26.7–32.0)	29.4 (26.0–31.3)
Maternal weight Median (IQR)	63.9 (57.0–79.0)	64.7 (58.8–73.1)	68.6 (60.8–75.0)
Parity = 0 *n* (%)	16 (64.0)	16 (61.5)	111 (66.5)
Previous preterm delivery *n* (%)	3 (12.0)	3 (11.5)	20 (12.0)
IVF *n* (%)	0 (0)	2 (7.7)	3 (1.7)

Maternal age: Kruskal–Wallis Test: *p* = 0.131

Gestational age: ANOVA: *p* = 0.044, Tukey post hoc Test: group 1 vs. 2: *p* = 0.290, 1 vs. 3: *p* = 0.034, 2 vs. 3 *p* = 0.913

Maternal weight: Kruskal–Wallis Test *p* = 0.801

Parity: chi-square *p* = 0.854

Previous preterm delivery: chi-square *p* = 0.946

IVF: chi-square *p* = 0.658

**Fig. 3:**
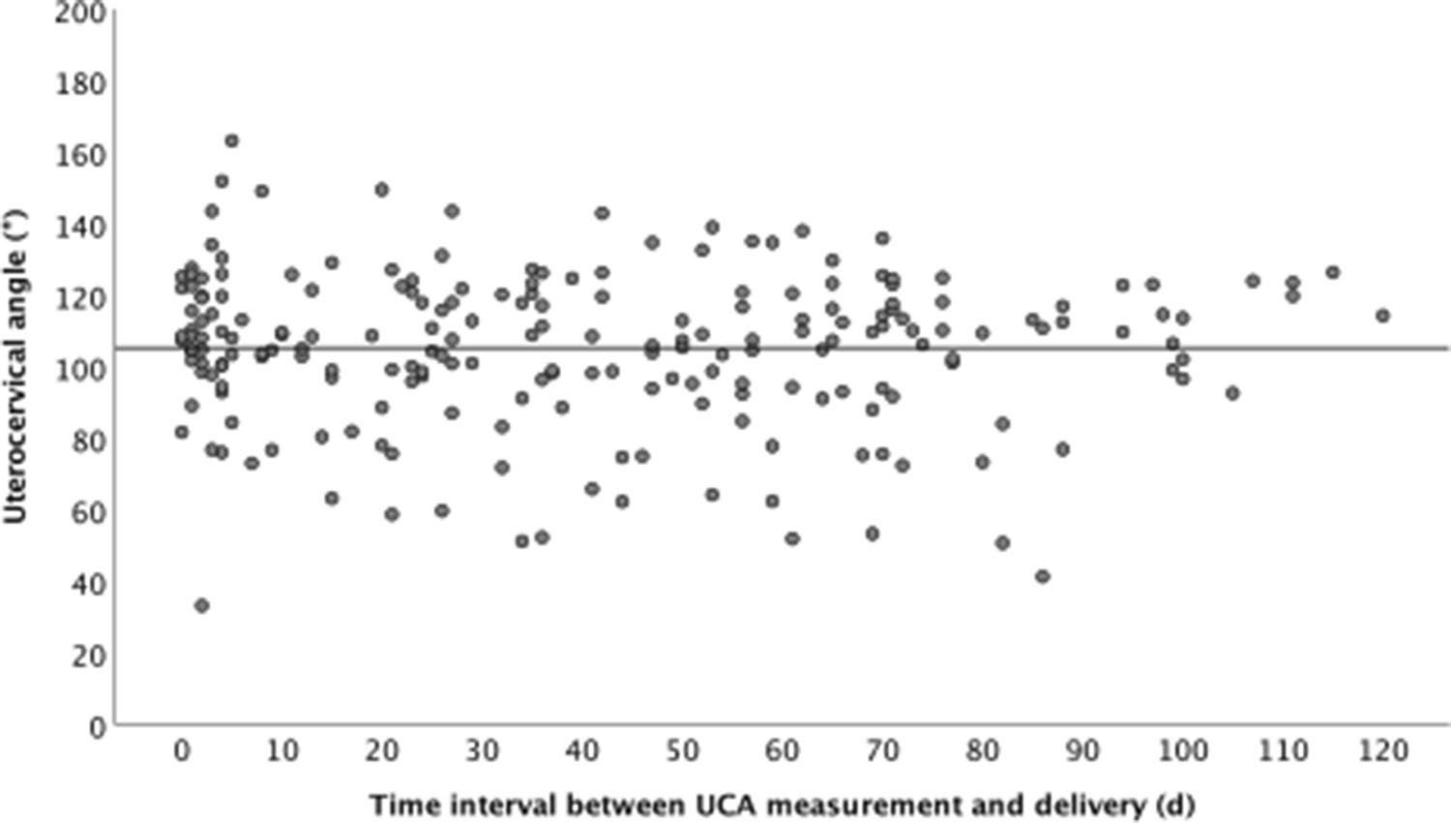
Time interval between measurement of the uterocervical angle (UCA) and delivery

The distribution of UCA measurements is shown in Fig. 3. Table 2 demonstrates the median cervical length, the proportion of cases with funneling and sludge and the UCA measurement in the three groups, delivery within 2 days, between 3–7 days and after 7 days. The UCA measurement was not helpful to distinguish between these three groups [median UCA measurements: 108.5°, 108.0°, and 107.3° (Kruskal–Wallis test *p* = 0.576)]. There was a significant difference in the proportion of cases with funneling in the three groups, but interestingly, there were more women with funneling who delivered between 3–7 days than within the first 2 days.

**Table 2:** Ultrasound assessment of the cervix

	Delivery ≤ 2 days	Delivery 3–7 days	Delivery ≥ 8 days
Cervical length Median (IQR)	12.0 (8.5–14.8)	13.0 (10.8–16.0)	18.0 (12.0–22.4)
Uterocervical angle Median (IQR)	108.5 (104.1–119.8)	108.0 (94.4–125.9)	107.3 (93.9–119.6)
Presence of funneling *n* (%)	13 (52.0)	16 (76.2)	76 (45.5)
Presence of sludge *n* (%)	0 (0)	3 (14.3)	9 (5.4)

Cervical length: Kruskal–Wallis Test: *p* < 0.0001, Dunn-Bonferroni post hoc Test: group 1 vs. 2: *p* = 1.000, 1 vs. 3: *p* < 0.0001, 2 vs. 3 *p* = 0.012

Uterocervical angle: Kruskal–Wallis Test: *p* = 0.576

Funneling: chi-square: *p* = 0.029

Sludge: chi-square: *p* = 0.315

We used linear regression analysis to identify correlations between the UCA measurement and the maternal and pregnancy characteristics as well as the cervical length and the presence of funneling and sludge). Only the presence of funneling had a significant impact on the UCA (presence of funneling *p* = 0.038, *r* = 0.142; maternal age *p* = 0.167; gestational age *p* = 0.857; parity *p* = 0.710, maternal weight *p* = 0.229; cervical length *p* = 0.602; presence of sludge *p* = 0.568: time interval measurement UCA measurement and delivery *p* = 0.619).

Uni- and multivariate logistic multiple regression analysis demonstrated that the delivery within 2 days was only dependent on the gestational age and the cervical length at the time of presentation (Table 3).

**Table 3:** Univariate and multivariate logistic regression to predict preterm delivery within 2 days

	Univariate		Multivariate	
	OR	*p*	OR	*p*
Maternal age	1.075	0.066		
Gestational age	**1.239**	**0.016**	**1.250**	**0.011**
Maternal weight	1.004	0.679		
Parity	0.922	0.803		
Previous preterm delivery	0.972	0.965		
Cervical length	**0.871**	** < 0.0001**	**0.863**	** < 0.0001**
Uterocervical angle	1.006	0.576		
Presence of funneling	0.885	0.774		
Presence of sludge	Infinitive	0.999		

The significant differences in the regression analysis is highlighted in bold

## Discussion

In this study, we have analyzed the role of the UCA in patients with threatened preterm labor and a cervical length of 25 mm or less. We have found, that the UCA cannot be used either as a primary nor as an additional marker to predict preterm birth in the next few days in women with preterm contractions.

In terms of the results of the multivariate regression analysis, our results are consistent with numerous previous studies indicating that the cervical length and the gestational age at the time of admission into the hospital are independent predictors for preterm delivery [3, 5, 7, 13–15].

So far, studies on the UCA focus on asymptomatic women in the second trimester. There is some evidence, that a wider UCA is associated with preterm birth before 34 weeks of gestation [11, 16]. Daskalakis et al. [11] reviewed the current body of literature and reported on 11 studies including about 3,000 women. The authors concluded, that second trimester UCA measurements could be a useful measurement in the prediction of preterm birth before 34 weeks. The most commonly reported cut-off measurements were 105° and 95° [11].

Battarbee et al. compared the impact of several ultrasound and obstetric characteristics—among them the UCA—in women with a cerclage that was placed up to 25 weeks. In 43% of the women, the cerclage was either ultrasound- or exam-indicated. The authors found several differences in the group of women who delivered prior or after 34 weeks. Most of these differences involved the length and the appearance of the cervix as well as the position of the cerclage. The UCA was not significantly different between the two groups [17].

The most relevant ultrasound parameter for the prediction of delivery within the next few days in women with preterm delivery remains the cervical length. Several studies have focused on this parameter and have proven its effectiveness in different conditions such as singleton and twin pregnancies and symptomatic and asymptomatic women [3, 6, 7, 14, 15]. Most commonly, a cut-off of 15 mm is used to stratify the further management of these pregnancies [7, 18, 19].

Further sonographic markers such as funneling or amnion fluid sludge have also been investigated to improve the prediction of preterm birth.

Saade et al. examined the impact of these findings in women between 16 and 22 weeks’ gestation with a cervical length of less than 30 mm. 17% and 5% of the women had either funneling or intra-amniotic sludge. In a multivariate regression analysis, the risk for preterm birth prior to 34 weeks was associated with the length of the cervix and sludge but not with funneling [20]. In symptomatic women, premature labor was significantly associated with the presence of funneling. However, in a prospective cohort of 200 women hospitalized for premature labor, a cervical length < 30 mm was significantly associated with the risk of preterm delivery with an adjusted OR of 3.9, but the presence of a funnel was not significantly associated with this risk [21, 22].

Espinoza et al. examined the relevance of slugde in women with preterm contractions [23]. In these cases, sludge was seen in about 23%. Regression analysis indicated that slugde was a significant predictor for delivery within 48 h and 7 days [23].

There are several other ultrasound markers that have been examined to assess the risk of preterm delivery in symptomatic women such as the dilatation of the cervical canal, the visibility of the membranes, the appearance of the gland area, the perfusion of the lower uterine segment and whether the canal was straight or curved [24, 25]. Unfortunately, the results were not supportive of these markers. Shear-wave elastography was considered useful as this method could potentially measure the stiffness of the cervix. However, the results were also not convincing enough to implement this tool into clinical practice [26].

Recently, Volpe et al. proposed to examine the sliding sign as an indirect marker of the cervical stiffness. They pushed gently on the cervix with the transvaginal probe and examined the dynamic changes of the cervix. The sign was present if sliding of the anterior cervical lip on the posterior one was observed. In women with a cervical length between 10 and 20 mm, the sign was present in 45% of the women who delivered within the subsequent 7 days and in only 15% in those who did not [27].

Our study has few limitations. The main limitation of the study is the retrospective design and the smaller sample size compared to the study from Dziadosz et al. [10]. Furthermore, the measurement of an angle carries a higher intra- and interobserver variability compared to a measurement of a distance such as the cervical length. Others have demonstrated that the measurement can be carried out with an acceptable reproducibility but we have learnt during the preparation of the study that training is necessary to standardize the measurement. This is particularly the case if there is funneling as the tangential placement of the ray on the lower uterine segment is challenging [10] This is nicely highlighted in Figs. 1 and 2.

In conclusion, in this study, we demonstrated that the measurement of UCA is not useful in predicting preterm birth in the subsequent 7 days after an episode of preterm contractions.

## Data Availability

The data are available on personal request.

## Code availability

Microsoft Word and Excel, SPSS.
